# Vγ9Vδ2 T Cells in the Bone Marrow of Myeloma Patients: A Paradigm of Microenvironment-Induced Immune Suppression

**DOI:** 10.3389/fimmu.2018.01492

**Published:** 2018-06-25

**Authors:** Barbara Castella, Myriam Foglietta, Chiara Riganti, Massimo Massaia

**Affiliations:** ^1^Laboratorio di Immunologia dei Tumori del Sangue (LITS), Centro Interdipartimentale di Ricerca in Biologia Molecolare (CIRBM), Università degli Studi di Torino, Turin, Italy; ^2^SC Ematologia, AO S. Croce e Carle, Cuneo, Italy; ^3^Dipartimento di Oncologia, Università degli Studi di Torino, Turin, Italy

**Keywords:** Vγ9Vδ2 T cells, immune checkpoints, multiple myeloma, immune suppression, bone marrow

## Abstract

Vγ9Vδ2 T cells are non-conventional T cells with a natural inclination to recognize and kill cancer cells. Malignant B cells, including myeloma cells, are privileged targets of Vγ9Vδ2 T cells *in vitro*. However, this inclination is often lost *in vivo* due to multiple mechanisms mediated by tumor cells and local microenvironment. Multiple myeloma (MM) is a paradigm disease in which antitumor immunity is selectively impaired at the tumor site. By interrogating the immune reactivity of bone marrow (BM) Vγ9Vδ2 T cells to phosphoantigens, we have revealed a very early and long-lasting impairment of Vγ9Vδ2 T-cell immune functions which is already detectable in monoclonal gammopathy of undetermined significance (MGUS) and not fully reverted even in clinical remission after autologous stem cell transplantation. Multiple cell subsets [MM cells, myeloid-derived suppressor cells, regulatory T cells, and BM-derived stromal cells (BMSC)] are involved in Vγ9Vδ2 T-cell inhibition *via* several immune suppressive mechanisms including the redundant expression of multiple immune checkpoints (ICPs). This review will address some aspects related to the dynamics of ICP expression in the BM of MM patients in relationship to the disease status (MGUS, diagnosis, remission, and relapse) and how this multifaceted ICP expression impairs Vγ9Vδ2 T-cell function. We will also provide some suggestions how to rescue Vγ9Vδ2 T cells from the immune suppression operated by ICP and to recover their antimyeloma immune effector functions at the tumor site.

## Introduction

Vγ9Vδ2 T cells have gained a solid reputation in cancer immunotherapy for their capacity to bridge innate and adaptive immunity and to participate to a multifaceted array of direct and indirect antitumor immune responses (1). Hematological malignancies, and especially B-cell malignancies, are privileged targets of Vγ9Vδ2 T-cell recognition and killing (2). This intrinsic susceptibility is due to the enhanced cell surface expression of stress-induced self-ligands and to the intense production of phosphorylated metabolites generated by the mevalonate (Mev) pathway. Isopentenyl pyrophosphate (IPP) is the prototypic Mev metabolite recognized by Vγ9Vδ2 T cells *via* TCR in association with the isoform A1 of the butyrophilin-3 (BTN3A1) protein family (3, 4). IPP is structurally related to the phosphoantigens generated by bacteria and stressed cells that are patrolled by Vγ9Vδ2 T cells as part of their duty to act as first-line defenders against infections and stressed cell at risk of malignant transformation (5).

One strategy commonly used *in vivo* and *in vitro* to activate Vγ9Vδ2 T cells is the stimulation of tumor cells, monocytes, and dendritic cells (DC) with aminobisphosphonates (NBP) like pamidronate and zoledronate (ZA) (6). These drugs inhibit farnesylpyrophosphate synthase in the Mev pathway (7) leading to intracellular IPP accumulation and extracellular IPP release which is sensed by Vγ9Vδ2 T cells *via* TCR and BTN3A1 (8). Wilhelm and colleagues (9) were the first to demonstrate that activation of Vγ9Vδ2 T cells with pamidronate and low-dose interleukin 2 (IL-2) could induce clinical responses in patients with B-cell lymphomas and multiple myeloma (MM). The ability of peripheral blood (PB) Vγ9Vδ2 T cells to proliferate *in vitro* after stimulation with pamidronate and IL-2 was a predictor of clinical response. A clinical trial of adoptively transferred *ex vivo* activated Vγ9Vδ2 T cells in combination with ZA and IL-2 was well tolerated, but showed very limited clinical efficacy (9). Additional studies in solid tumors have also fallen short of clinical expectations (10–13). Understanding why Vγ9Vδ2 T cells perform so poorly when intentionally recruited *in vivo* or *ex vivo* to kill tumor cells is mandatory to really exploit their antitumor properties. One possible explanation is that activated Vγ9Vδ2 T cells do not reach the tumor site or, if reached, they are overwhelmed by the immune suppressive contexture operated by tumor cells and neighboring cells in the tumor microenvironment (TME).

The TME is the protective niche which helps tumor cell to resist chemotherapy and escape immune surveillance (14). Although immune effector cells are often recruited in the TME by the tumor mutational load and the inflammatory milieu, their antitumor functions are blunted by direct or indirect inhibitory signals generated by tumor cells and neighboring cells in the TME (15). Vγ9Vδ2 T cells are not exempted from this immune suppressive contexture operated *via* soluble and cellular factors (16). Soluble factors include transforming growth factor-β, prostaglandins, and kynurenins (17–19). Cellular factors include regulatory T cells (Tregs), myeloid-derived suppressor cells (MDSC), bone marrow-derived stromal cells (BMSC), and others. The discovery that immune checkpoints (ICPs) and their ligands (ICP-L) are abundantly expressed by tumor cells, immune effector cells, and immune suppressive cells have helped to understand the mechanisms promoting the immune suppressive cross talk in the TME and provided new opportunities of interventions.

In this review, we will discuss how the ICP/ICP-L circuitry undermines Vγ9Vδ2 T-cell function and how Vγ9Vδ2 T cells are very early and sensitive detectors of the TME immune suppressive contexture in MM patients. Lessons learned from Vγ9Vδ2 T cells in MM can be instrumental to improve Vγ9Vδ2 T-cell-based immunotherapy in cancer.

## The Immune Suppressive TME in Myeloma

Multiple myeloma is a prototypic disease where malignant myeloma cells actively remodel the bone marrow (BM) microenvironment to establish a protective niche to support their growth, immune evasion, and drug resistance. MM is invariably preceded by a precursor asymptomatic stage of monoclonal gammopathy of undetermined significance (MGUS) with an estimated risk of progression to symptomatic disease ranging from less than 1% to more than 3% per year. This range depends on risk factors traditionally ascribed to intrinsic features of myeloma cells. Genomic alterations determining clonal advantage are already detectable in MGUS indicating that the probability of progression is also dependent on extrinsic factors such as the composition of the surrounding TME (20). TME in MGUS and MM consists of a non-cellular component, the extracellular matrix, and of a heterogeneous cellular compartment that includes hematopoietic and non-hematopoietic cells. Both the non-cellular and cellular components are edited by myeloma cells to elude immune surveillance and insure their undisturbed survival and progressive expansion (14, 15, 21).

Immune escape in the BM of MGUS and MM patients is achieved by shifting the balance between immune effector and immune suppressor functions as in many other cancers. The immune suppressive mechanisms include the local recruitment and/or activation of immune suppressor cells like Tregs, MDSC, the protumoral polarization of tumor-associated macrophages and/or mesenchymal stem cells, and the differentiation and activation of Th17 cells (22). The wane of immune effector functions includes impaired phagocytosis, ineffective antigen presentation, and T-cell costimulation by DC, B-cell defects and humoral deficiencies, and NK and NKT cell dysfunctions (23). The protumoral immune shift in the TME is driven by soluble factors and cellular interactions, including the recently discovered ICP/ICP-L circuitry.

**Figure 1:**
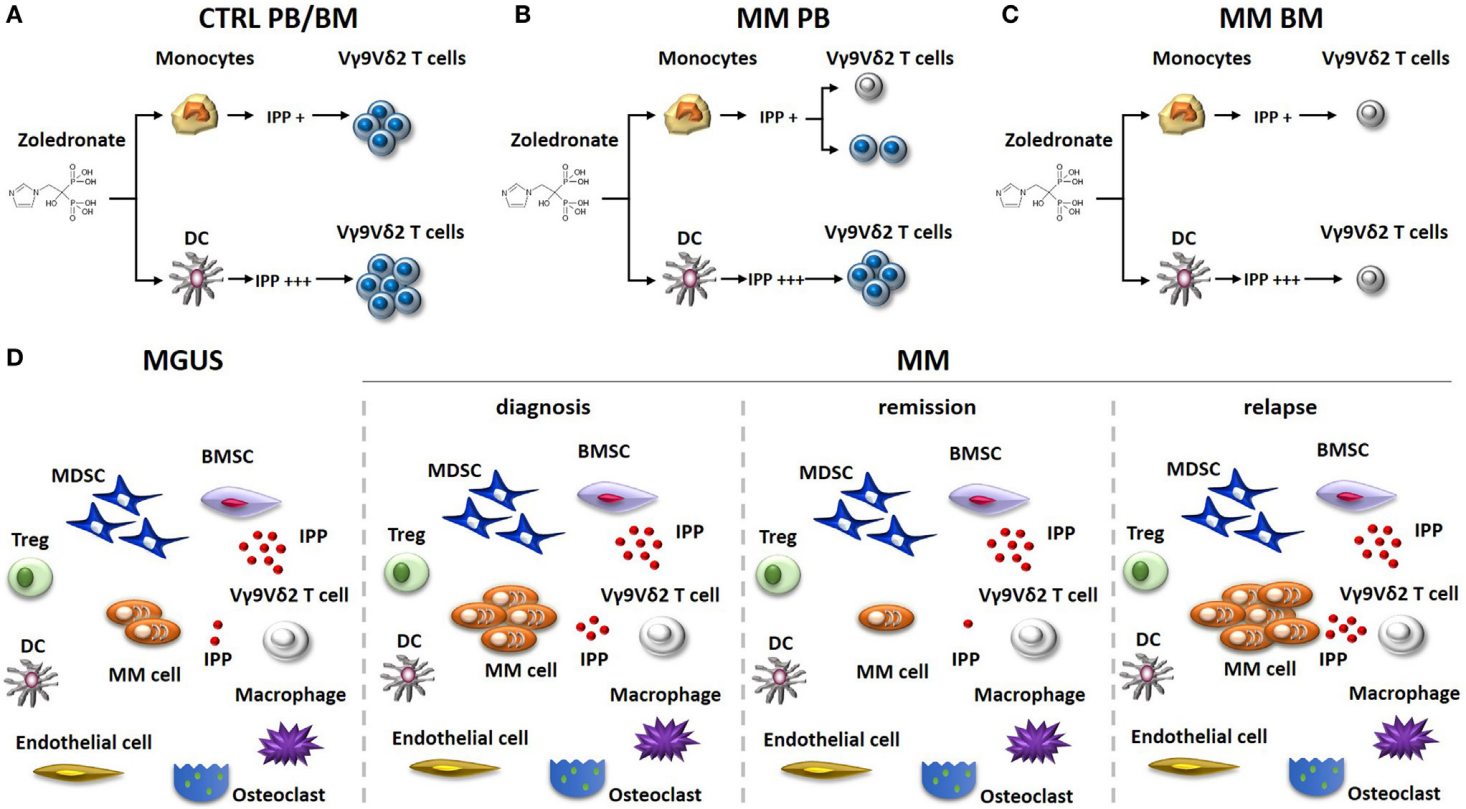
Vγ9Vδ2 T-cell impairment in the immune suppressive TME of MM patients. The stimulation of monocytes or DC with zoledronate (ZA) and low dose of IL-2 is commonly used *in vitro* to induce IPP-dependent Vγ9Vδ2 T-cell activation. **(A)** This approach is uniformly effective in healthy donors (Ctrl), no matter whether Vγ9Vδ2 T cells are from the PB or the BM. ZA-treated DC are more effective than ZA-treated monocytes to induce Vγ9Vδ2 T-cell activation because they produce higher IPP amounts. **(B)** PB Vγ9Vδ2 T cells are refractory to ZA stimulation (gray colored) in about 50% of MM patients at diagnosis, when monocytes are used as stimulators. Anergy in these patients can be recovered if ZA-treated DC instead of monocytes are used as stimulators. **(C)** BM Vγ9Vδ2 T cells from MM patients are uniformly anergic to ZA stimulation and the anergy is not corrected by ZA-treated DC. **(D)** The immune suppressive TME of MGUS and MM patients includes suppressor cells like Tregs, MDSC, and BMSC (that produce high amounts of IPP). The proportion of these cells is very similar irrespective of the disease status. MDCS are PD-L1^+^ in MGUS and MM irrespective of the disease status and release high amounts of IPP in the TME. Other cells playing a major role in the TME are macrophages, endothelial cells, and osteoclasts. The proportion of these cells is variable according to diagnosis and disease status. The defective pAg reactivity of Vγ9Vδ2 T-cell (gray colored) is present in MGUS, MM at diagnosis, MM in remission, and MM in relapse. Elimination of myeloma cells (that are PD-L1^+^ and release IPP) is insufficient in the short/intermediate term to recover Vγ9Vδ2 T-cell functions. It is possible that the persistence of PD-L1^+^ cells in the TME (i.e., MDSC), high levels of extracellular IPP, and PD-1 expression (and other ICP) on their cell surface continue to render functionally incompetent BM Vγ9Vδ2 T cells even in the remission phase. Abbreviations: DC, dendritic cells; ZA, zoledronate; IPP, isopentenyl pyrophosphate; PB, peripheral blood; BM, bone marrow; Ctrl, healthy subjects; MM, multiple myeloma; Tregs, regulatory T cells; MDSC, myeloid-derived suppressor cells; BMSC, bone marrow-derived stromal cells; MGUS, monoclonal gammopathy of undetermined significance; TME, tumor microenvironment; ICP, immune checkpoint; PD-1, programmed cell death protein 1.

For many years, the conventional wisdom has been that the immune balance is tipped in favor of myeloma cell control in MGUS and early stages of MM, whereas the balance is tipped in favor of myeloma cell growth in advanced disease. The wisdom was based on the results obtained from experiments exploring the phenotype and function of T cells, NK cells, and NKT cells in MGUS and early MM stages compared to advanced MM stages (24–26). Nowadays, it is clear that the TME of MGUS subjects already harbor a number of immune dysfunctions. The functional interrogation of pAg reactivity of Vγ9Vδ2 T cells in MGUS and MM patients at different stages of the disease (diagnosis, remission, and relapse) has been particularly enlightening (Figure 1). We have previously shown that Vγ9Vδ2 T cells from approximately 50% of MM patients are anergic to ZA stimulation at diagnosis when this assay is performed in PB and monocytes are used to generate IPP (27) (Figure 1B). The anergy is reversible if ZA-treated DC, and not monocytes, are used to stimulate PB Vγ9Vδ2 T cells, one possible explanation being that the higher IPP production by DC after ZA stimulation (28, 29). The proportion of anergic MM patients increases to 80–90% if ZA stimulation is carried out in the BM using monocytes as IPP-presenting cells. Unlike PB, the strategy to use ZA-treated DC to recover Vγ9Vδ2 T-cell proliferation in the BM is ineffective (Figure 1C), and neither the removal nor the functional inhibition of suppressive cells like Tregs or MDSC are sufficient to recover BM Vγ9Vδ2 T-cell proliferation (30). Crossover experiments have clearly shown that the defective pAg reactivity is peculiar to BM Vγ9Vδ2 T cells, which do not proliferate no matter whether they are stimulated with BM- or PB-derived ZA-treated DC. Vγ9Vδ2 T-cell anergy is already detectable in the BM of MGUS individuals, largely anticipating the dysfunction of T and NKT cells. BM Vγ9Vδ2 T cells remain anergic to pAg stimulation also in MM patients who are in remission after autologous stem cell transplantation (30) (Figure 1D). Altogether, these data indicate that Vγ9Vδ2 T cells are unique among other immune effector cells in sensing the very early and persistent immune suppressive TME commitment in MGUS and MM.

## Programmed Cell Death Protein 1 (PD-1)/PD-L1 Network Impairs Vγ9Vδ2 T-Cell Activation in the TME

Immune checkpoints/ICP-L are expressed by a variety of immune cells to control the strength and duration of immune responses and maintain T-cell homeostasis and self-tolerance (31). Smartly, tumor cells have learned very quickly how to hijack the ICP/ICP-L circuitry to withstand immune recognition and onslaught. Cytotoxic T-lymphocyte-associated antigen 4 (CTLA4) and PD-1 are so far the ICP more often targeted for therapeutic purposes (32). PD-1 is expressed on the surface of dysfunctional T and B cells and inhibits T cell-mediated apoptosis after engagement by PD-L1 expressed by tumor cells (33–36). The devilish plot to protect tumor cells from immune recognition and killing begins in the tumor-draining lymph nodes where the PD-1/PD-L1 axis interferes with antigen presentation to blunt the activation of tumor-specific immune responses (37, 38). The inhibition of adaptive immune responses in secondary lymphoid organs is strategically implemented in the TME by ICP/ICP-L-dependent blockade of antitumor responses mediated by innate immunity (i.e., NK cells and Vγ9Vδ2 T cells) (30, 39). Based on these premises, ICP blockade (mainly pursued using anti-CTLA4 and/or anti-PD-1/PD-L1 mAbs) has been granted FDA approval in solid tumors and Hodgkin lymphoma (40, 41).

In the context of MM, increasing evidences suggest that the PD-1/PD-L1 pathway plays an active role in the generation of the immunosuppressive TME (42, 43). Myeloma cells offer high levels of PD-L1 to PD-1-expressing T and NK cells in the TME (43–45), and *in vitro* studies showed enhanced myeloma cell killing by T and NK cells after PD-1 and/or PD-L1 blockade (46). In a mouse model, Hallett and colleagues demonstrated that PD-L1 expression in myeloma cells decreases cytotoxic function, cytokine production, and proliferation of PD-1^+^ T and NK cells leading to their functional exhaustion (47). Consistent with this observation, Paiva et al. reported a prolonged survival in disseminated myeloma-bearing mice after PD-1 blocking (42), corroborating the therapeutic exploration of PD-1 blockade in MM.

However, unsatisfactory results of single agent anti PD-1 mAb failed to meet the expectations in the clinical setting. Combination approaches with immunomodulatory drugs (lenalidomide or pomalidomide) and dexamethasone proved synergistic effects in phase I/II trials, nurturing hopes for therapeutic exploitation of PD-1 blockade in MM (48–50). Immunotherapy with daratumumab is currently under investigation as an alternative partner to improve efficacy of PD-1 blockade in a multiphase randomized clinical trial (NCT03357952) recruiting relapsed refractory myeloma patients. Only a deeper understanding of molecular mechanisms triggered by PD-1/PD-L1 signaling pathway may lead to rationally identify targeted strategies to overcome resistance to PD-1 blockade.

Although the function of PD-1 has been extensively studied in mouse and human conventional αβ T cells (51–53), little is known about the role of PD-1/PD-L1 signaling in human Vγ9Vδ2 T cells. Iwasaki et al. analyzed PD-1 expression in PB Vγ9Vδ2 T cells after pAg stimulation in healthy donors and breast cancer patient (54). They found that PD-1^+^ Vγ9Vδ2 T cells in breast cancer patients produced less IFNγ had lower cytotoxic activity and CD107 degranulation than PD-1^−^ cells after challenging with PD-L1^+^ tumor target cells. Zumwalde et al. (55) have stressed the different kinetics of PD-1 expression in normal Vγ9Vδ2 T cells upon pAg stimulation compared with tumor-experienced Vγ9Vδ2 T cells. BM Vγ9Vδ2 T cells from myeloma patients represent a paradigmatic example of functionally impaired tumor-experienced Vγ9Vδ2 T cells. PD-1 expression in Vγ9Vδ2 T cells from normal donors peaks approximately 3–4 days after pAg stimulation afterward PD-1 expression returns to baseline values (55). This is very different compared with myeloma patients in which PD-1 expression increased in BM anergic Vγ9Vδ2 T cells after ZA stimulation, suggesting that these cells are intrinsically programmed to increase their threshold of refractoriness to pAg-induced TCR stimulation *via* PD-1 upregulation (30). Interestingly, PD-1 expression in myeloma BM Vγ9Vδ2 T cells is predominant in the central memory subset, which in normal conditions is the subset with the highest proliferative capacity to pAg stimulation (30).

One possible mechanism to explain PD-1 expression in BM Vγ9Vδ2 T cells, already detectable in MGUS when the myeloma cell infiltration is still low (<10% by definition), is the prolonged TCR engagement by pAg in the TME. Preliminary results from our lab indicate that myeloma cells are not the only IPP producers in the TME, and that BMSC in MGUS and MM also produce and release very high amounts of IPP in the extracellular microenvironment (8) (Figure 1D). Thus, it is possible that a chronic TCR engagement within an immune suppressive TME, characterized by inappropriate costimulatory signals and/or cytokines, leads to PD-1 expression and functional exhaustion of Vγ9Vδ2 T cells.

Our study has been the first to show that human MDSC are PD-L1^+^ in the TME suggesting that this is an additional mechanism exploited by these cells to exert local immune suppression against PD-1^+^ effector cells. Interestingly, the BM is highly hypoxic in MM (56) and experimental data in tumor-bearing mice have shown that the hypoxia-inducible factor-1α selectively upregulates PD-L1 in tumor-infiltrating MDSC, but not in MDSC from peripheral lymphoid organs (57). Hypoxia has been reported to increase the immune suppressive TME contexture *via* upregulation of a variety of ICP/ICP-L (58). Extracellular adenosine, which accumulates due to tissue hypoxia, also contributes to ICP/ICP-L upregulation (59), and adenosine levels are significantly higher in the BM of myeloma due to the highly coordinated expression of adenosinergic ecto-nucleotidases (CD39/CD73/CD38/CD203a) strategically located at the interface between myeloma cells and neighboring cells (60). Preliminary data from our lab indicate that BMSC, another major protumoral component in the BM niche of MGUS and MM patients, are PD-L1^+^, further confirming that there is a redundancy of immune suppressor cells exploiting the ICP/ICP-L circuitry to hamper myeloma cell recognition and elimination by immune effector cells in the TME. The finding that BM Vγ9Vδ2 T cells are PD-1^+^ in MGUS, MM at diagnosis, and even in remission, confirms the unique sensitivity of these cells to the immune suppressor imprinting operated by the TME which is not overcome even when myeloma cells have been cleared from the BM. One possible explanation is that the immune suppression is exerted by PD-L1^+^ cells other than myeloma cells, like MDSC and BMSC, whose percentages and PD-L1 expression remain unchanged in the BM of MM in remission (30) (Figure 1D).

## Strategies to Rescue Antitumor Vγ9Vδ2 T-Cell Function in the TME: Lessons from MM

**Figure 2:**
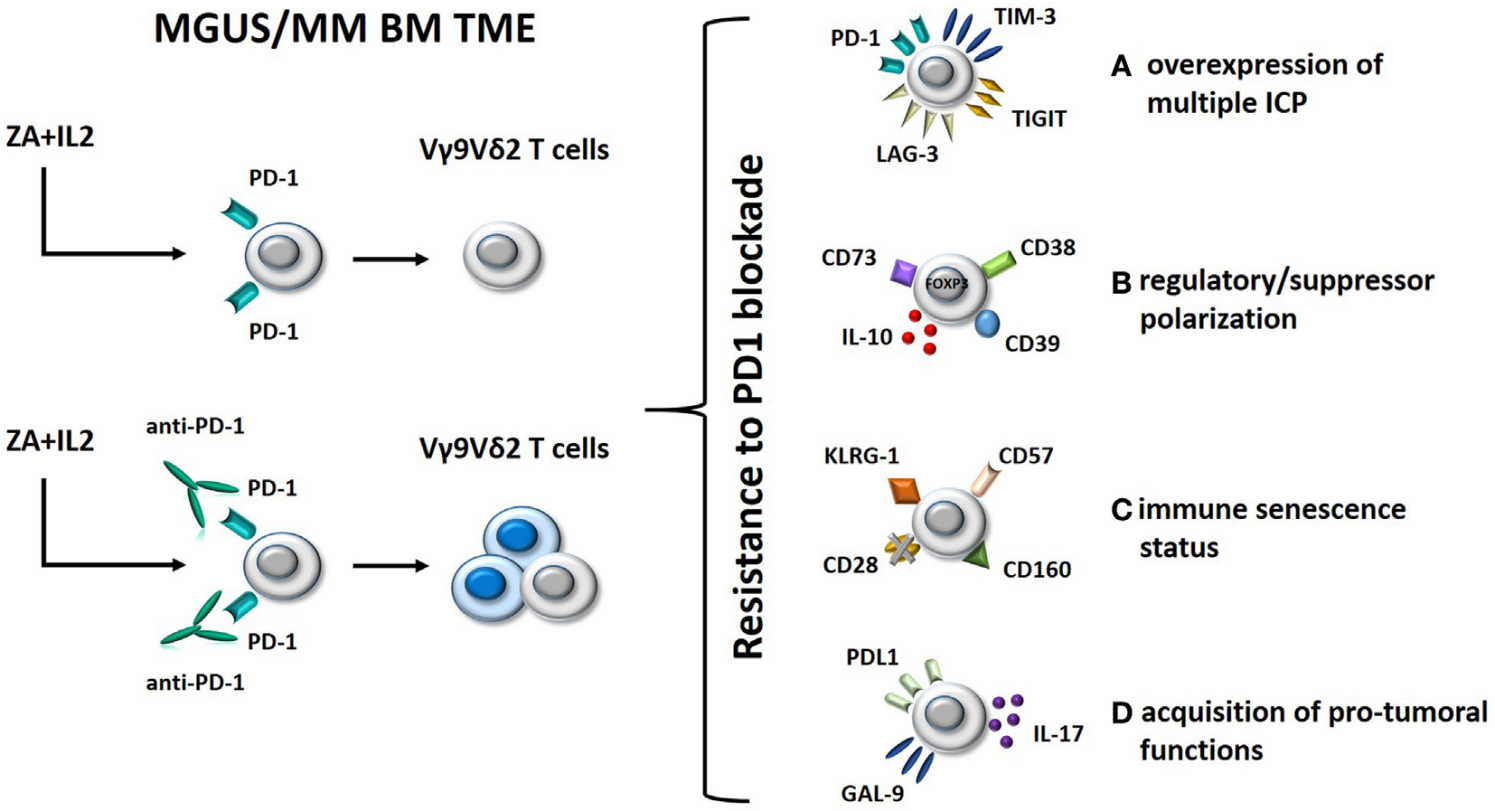
Hypothetical mechanisms of resistance to PD-1 blockade in BM Vγ9Vδ2 T cells from MM patients. Left panel: BM Vγ9Vδ2 T cells in MGUS and MM are anergic to ZA + IL-2 stimulation; this anergy is only partially rescued by single agent PD-1 blockade. Right panel: possible mechanisms of resistance to immune recovery triggered by single agent PD-1 blockade. (A) Alternative inhibitory ICPs, such as TIM-3, TIGIT, and LAG-3, are expressed by BM Vγ9Vδ2 T cells to reinforce their anergy and resist to PD-1 blockade; (B) Vγ9Vδ2 T cells have been functionally polarized to express inhibitory molecules (FOXP3, CD73, CD39, and CD38) and release immune suppressive cytokines (IL-10) as a consequence of their long-term exposure to tumor cells in the BM; (C) BM Vγ9Vδ2 T cells have acquired a senescence status (CD57^+^CD160^+^KLRG-1^+^CD28^−^ phenotype) which is hard to reverse by single PD-1 blockade; (D) PD-1 engagement of anergic/senescent BM Vγ9Vδ2 T cells induces the expression of PD-L1 and galectin-9 (GAL-9), and IL-17 secretion leading to immune suppressive effects on other effector cells like CD8^+^ cells. Abbreviations: MGUS, monoclonal gammopathy of undetermined significance; MM, multiple myeloma; BM, bone marrow; TME, tumor microenvironment; ZA, zoledronate; IL-2, interleukin 2; ICP, immune checkpoint; TIM-3, T-cell immunoglobulin and mucin-domain containing-3; LAG-3, lymphocyte-activation gene 3; PD-1, programmed cell death protein 1.

Clinical trials using anti-PD-1 mAbs as single agents in MM have failed to confirm the excellent premises of experimental data (61, 62). Interestingly, we have shown that single agent PD-1 blockade is insufficient to fully recover the antitumor activity of BM Vγ9Vδ2 T cells in MM (30). Thus, Vγ9Vδ2 T cells are excellent tools to decipher the mechanisms developed by Vγ9Vδ2 T cells and other immune effector cells to resist immune recovery triggered by ICP/ICP-L blockade in the TME (Figure 2). Understanding these mechanisms of resistance is important to improve the efficacy of immune interventions based on ICP/ICP-L blockade in MM and other cancers.

One mechanism could be the expression of alternative ICP under resting conditions or after pAg stimulation and/or PD-1 blockade (Figure 2, option A). Double PD-1 and T-cell immunoglobulin and mucin-domain containing-3 (TIM-3) expression in tumor-infiltrating lymphocytes from tumor-bearing mice identifies the most dysfunctional CD8^+^ T cells in the TME of these mice, and concurrent PD-1 and TIM-3 blockade significantly improves the antitumor activity of these cells, much better than single inhibition (63). The expression of multiple ICP/ICP-L expression on individual immune cells and tumor cells has recently been proposed as a mechanism of acquired resistance to single PD-1 blockade also in human cancer (64, 65). TIM-3, lymphocyte-activation gene 3 (LAG-3), and TIGIT are examples of alternative ICP that could be expressed on the cell surface of BM Vγ9Vδ2 T cells and could restrain the efficacy of single PD-1 blockade. Targeting multiple ICP could be an attractive strategy to improve the recovery of antitumor Vγ9Vδ2 T-cell responses (Figure 2, option A).

Another mechanism could be the regulatory/suppressor polarization of BM Vγ9Vδ2 T cells driven by the TME (66, 67). This functional polarization cannot be reverted by single PD-1 blockade. Vγ9Vδ2 T cells with regulatory functions have initially been described by Casetti et al. (68), who reported the *in vitro* induction of FOXP3^+^ regulatory Vγ9Vδ2 T cells after pAg stimulation in the presence of TGF-β1 and IL-15. Other groups have confirmed the emergence of regulatory/suppressor Vγ9Vδ2 T cells as a consequence of pAg activation in the presence of selected cytokines (69, 70). Ma et al. have reported an increased proportion of regulatory Vγ9Vδ2 T cells in the PB of MM patients which could suppress antimyeloma immune responses with the same efficiency of conventional Tregs (71).

Single PD-1 blockade may not be sufficient to revert the regulatory/suppressor polarization of BM Vγ9Vδ2 T cells. Preliminary data in our lab indicate that PD-1 blockade of BM Vγ9Vδ2 T cells in MM could even worsen this polarization by inducing the expression of additional inhibitory molecules (FOXP3, CD73, CD39, and CD38) and the release of suppressive factors like IL-10 (Figure 2, option B). In this case, PD-1 blockade should be integrated by strategies aimed at preventing the detrimental BM Vγ9Vδ2 T-cell polarization and/or the regulatory/suppressor functions exerted by polarized Vγ9Vδ2 T cells in the TME.

Another major hurdle preventing the full recovery of antitumor Vγ9Vδ2 T-cell functions by PD-1 blockade could be their immune senescence status (Figure 2, option C). Immune senescence is the hallmark of oligoclonal T cells which accumulate in the PB of MM patients with progressive and advanced disease (72–74). The immune competence of senescent cells is very hard to resurrect by single PD-1 blockade. The CD57^+^CD160^+^KLRG-1^+^CD28^−^ phenotype might portray a distinct population of senescent Vγ9Vδ2 T cells gathered in the BM of MM patients which require multiple approaches to overcome resistance to PD-1 blockade.

Finally, the acquisition of protumoral functions by Vγ9Vδ2 T cells that are long-term resident in the TME could be another mechanism of resistance to single PD-1 blockade. The inappropriate expression of ICP-L such as PD-L1 and galectin-9 (GAL-9) in Vγ9Vδ2 T cells could affect *via* PD-1 and TIM-3 the antitumor responses of other immune effector cells in the TME (Figure 2, option D). Likewise, production of IL-17 by Vγ9Vδ2 T cells, as reported for selected γδ subsets in solid tumors (75), may contribute to reinforce the immune suppressive TME imprinting by recruiting MDSC (76) and polarizing neutrophils (77). This hypothetical scenario suggests that PD-L1/GAL-9 and IL-17 (or its receptor) could be novel targets to rescue antimyeloma Vγ9Vδ2 T-cell function.

Whether the mechanisms reported above are operative under baseline conditions or sharpened by pAg stimulation in the presence of PD-1 blockade is a matter of current investigation in our lab. Preliminary data suggest that the intracellular metabolic and signaling pathways evoked by PD-1 blockade could worsen the immune competence status of pAg-experienced Vγ9Vδ2 T cells.

## Conclusion

Vγ9Vδ2 T cells are programmed by default to behave as very effective professional killers of malignant B cells, including myeloma cells. We propose that Vγ9Vδ2 T cells are very precociously neutralized by myeloma cells in cooperation with neighboring cells in the TME of MGUS and MM patients. Finalistically speaking, it makes sense that myeloma cells inactivate or co-opt in their favor those immune cells mostly well suited to threaten their survival. This is done very early at the stage of MGUS and the uncontrolled production of IPP by BMSC and myeloma cells is probably a relevant initiating event.

Functional interrogation of BM Vγ9Vδ2 T cells from MM patients in remission has revealed that clearance of myeloma cells does not automatically imply the recovery of a fully immune competent TME.

ICP/ICP-L abundantly expressed by tumor cells, immune effector cells, and immune suppressive cells are major promoters of immune suppressive cross talks in the TME at any stage and hamper the antimyeloma activity of BM Vγ9Vδ2 T cells. Single agent PD-1 blockade is insufficient to fully recover the antitumor activity of Vγ9Vδ2 T cells *in vitro*, especially in MM at diagnosis or in relapse. These data indicate that additional immune suppressive mechanisms are involved in the anergy of Vγ9Vδ2 T cells. A working knowledge of these mechanisms may yield insight into the development of more effective interventions to fully exploit the immune potency of Vγ9Vδ2 T cells in MM and other cancers. This knowledge could be profitably implemented by next generation sequencing studies investigating the genetic and epigenetic consequences of cell-to-cell interactions of Vγ9Vδ2 T cells and other cell subsets in the TME of MGUS and MM patients.

## Author Contributions

All authors have made substantial contributions to text and figures and have approved the manuscript for submission.

## Conflict of Interest Statement

The authors declare that the research was conducted in the absence of any commercial or financial relationships that could be construed as a potential conflict of interest.
